# Transcranial Direct Current Stimulation for Post-Concussion Syndrome: Study Protocol for a Randomized Crossover Trial

**DOI:** 10.3389/fneur.2017.00164

**Published:** 2017-05-02

**Authors:** Robson Luis Oliveira de Amorim, André Russowsky Brunoni, Mirian Akiko Furutani de Oliveira, Ana Luiza Costa Zaninotto, Marcia Mitie Nagumo, Vinícius Monteiro de Paula Guirado, Iuri Santana Neville, Gláucia Rosana Guerra Benute, Mara Cristina Souza de Lucia, Wellingson Silva Paiva, Almir Ferreira de Andrade, Manoel Jacobsen Teixeira

**Affiliations:** ^1^Division of Neurosurgery, University of São Paulo Medical School, São Paulo, Brazil; ^2^Division of Psychiatry and Psychology, University Hospital of São Paulo University, São Paulo, Brazil; ^3^Division of Psychology, University of São Paulo Medical School, São Paulo, Brazil

**Keywords:** brain injuries, post-concussion syndrome, transcranial direct current stimulation, non-invasive brain stimulation, crossover studies

## Abstract

**Background:**

Mild traumatic brain injury (MTBI) represents 70–80% of all treated brain injuries. A considerable proportion of MTBI patients experience post-concussion symptoms for a prolonged period after MTBI, and these symptoms are diagnosed as persistent post-concussion syndrome (PPCS). PPCS is defined as a range of physical, cognitive, and emotional symptoms. However, memory and executive dysfunction seems to be one of the most debilitating symptoms. Recently, non-invasive brain stimulation has been studied as a potential treatment method for traumatic brain injury (TBI) patients. Therefore, our primary goal is to verify the effects of transcranial direct current stimulation (tDCS) in patients with PPCS who demonstrate cognitive deficits in long-term episodic memory, working memory, and executive function following MTBI.

**Methods/design:**

This is a randomized crossover trial of patients with a history of MTBI with cognitive deficits in memory and executive function. Thirty adult patients will be randomized in a crossover manner to receive three weekly sessions of anodal tDCS (2 mA) at left dorsolateral prefrontal cortex, left temporal cortex, and sham stimulation that will be performed at 7-day intervals (washout period). The clinical diagnosis of PPCS will be determined using the Rivermead Post-Concussion Symptoms Questionnaire. Patients who meet the inclusion criteria will be assessed with a neuropsychological evaluation. A new battery of computerized neuropsychological tests will be performed before and immediately after each stimulation. Statistical analysis will be performed to determine trends of cognitive improvement.

**Discussion:**

There is paucity of studies regarding the use of tDCS in TBI patients, and although recent results showed controversial data regarding the effects of tDCS in such patients, we will address specifically patients with PPCS and MTBI and no brain abnormalities on CT scan other than subarachnoid hemorrhage. Moreover, due to the missing information on literature regarding the best brain region to be studied, we will evaluate two different regions to find immediate effects of tDCS on memory and executive dysfunction.

**Clinical Trial Registration:**

www.ClinicalTrials.gov, identifier NCT02292589 (https://register.clinicaltrials.gov).

## Introduction

Traumatic brain injury (TBI) is the leading cause of death and disability among children and young adults. Approximately 90% of more than two million annual traumatic brain injuries in the United States are classified as mild traumatic brain injury (MTBI) (1). The criteria for clinical identification of MTBI consists of one or more of the following: a Glasgow Coma Scale (GCS) score of 13–15, confusion and disorientation, loss of consciousness for 30 min or less, posttraumatic amnesia for less than 24 h, and/or other transient neurologic abnormalities (2).

Mild traumatic brain injury has been referred to as a “silent epidemic” because the problems experienced by patients after injury are often unnoticed but can have profound consequences, such as long-term physical, mental, social, or occupational sequelae (3–5). For the majority of patients, MTBI follows a natural course in which the symptoms rapidly resolve within 3 months. However, a considerable proportion of patients with MTBI experience post-concussion symptoms (PCS) for a prolonged period after injury (6). The range of these symptoms can include headache, dizziness, fatigue, irritability, sleep disturbance, difficulties with concentration, memory loss, stress intolerance, light and sound sensitivity, balance problems, anxiety, and a depressed mood. Such prolonged post-injury effects are referred to as persistent post-concussion syndrome (PPCS).

The consequences of PPCS are overwhelming and include a broad spectrum of cognitive, behavioral, and sensorimotor disabilities that dramatically reduce the quality of life; therefore, PPCS is a worldwide public health problem that requires long-term care (7). Given the magnitude of the problem and the lack of specificity of PPCS symptoms, there is an obvious need for studies to examine whether early intervention might reduce the duration of PPCS symptoms.

### Neurological and Neuropsychological Findings concerning PPCS

Along with changes in emotional regulation, impairments in attention, memory, and executive function dominate the clinical profile of PPCS (8). However, a variety of symptoms can exist following concussion. The most common symptoms are a disruption of consciousness and a brief period of posttraumatic amnesia. The individual may also report feeling as though he or she is “in a fog.” Somatic symptoms, such as headache, fatigue, and balance problems, are also very common. During the acute stages following concussion, a patient may demonstrate disturbances in memory and concentration and feel “slowed down” (9).

It has been hypothesized that PPCS is caused by microstructural damage to the brain due to shearing injury, which is not detectable with conventional imaging techniques and may be responsible for functional deficits (10, 11). The brain regions affected by a concussion seem to especially involve the mesial regions and deeper regions including the hippocampus and corpus callosum. This “preference” would justify the deficits found in post-concussion patients who have memory complaints. Another area that is frequently involved is the prefrontal cortex, which would explain the executive function deficits that can persist even 3 months after the trauma (12, 13).

Cognitive dysfunction is characterized by impairments in attention, concentration, memory, and/or executive function. Patients may have difficulties following instructions and performing tasks or jobs that would have been routine before the trauma (14).

The rapid resolution of symptoms after MTBI raises questions of whether patients can directly benefit from neuropsychological interventions. However, PCS can undoubtedly persist in some cases. Addressing such cases through research in neuropsychology and neuroscience would help to improve our understanding of the progression and etiology of PCS, as well as produce new interventions to help patients who do not improve as expected (15).

Neuropsychological assessment provides diagnostic information about the nature and extent of cognitive dysfunction in neurological conditions, including MTBI. The National Institute of Mental Health and Neurosciences suggests that some neuropsychological batteries have adequate sensitivity and ecological validity to assess the cognitive deficits associated with MTBI (16).

### Cognitive Rehabilitation—The Role of Non-Invasive Neuromodulation

Recent reports have documented the therapeutic potential of non-invasive neuromodulation techniques for cognitive enhancement (17–23). The main techniques used for this purpose are repetitive transcranial magnetic stimulation (rTMS) and transcranial direct current stimulation (tDCS).

Transcranial direct current stimulation is a non-invasive neuromodulatory technique that is inexpensive, is easy to use, and applicable to the modification of cerebral excitability. This technique delivers weak polarizing direct current to the cortex *via* two electrodes placed on the scalp. One electrode is an active electrode that is placed over the targeted cortical region, whereas the second electrode is a reference electrode that is typically placed over the contralateral supraorbital area or a non-cephalic region (24). Several studies have demonstrated that a single session of rTMS or tDCS can improve performance on computerized neuropsychological tests that measure cognitive functions, such as working memory, verbal fluency, reaction time, cognitive interference, and sustained attention in patients with TBI (23, 25–28). These stimulation techniques appear to modulate not only “cold” (non-emotional) cognitive functions but also cognitive processes that involve decision-making, attention, and working memory, as shown in studies of patients with depression (29) and eating disorders (30, 31).

Most studies show that anodal instead of cathodal tDCS is better to enhance cognitive function (32–44). Repetitive anodal tDCS (A-tDCS) applied to the dorsolateral prefrontal cortex (DLPFC) was reported to enhance cognition (37–39), reduce depression (40), and suppress food craving (41). Some studies showed short-term facilitation effects on visual recognition memory and memory peformance after prefrontal and temporal A-tDCS applied 30 min at 2 mA, in patients with Alzheimer disease (42–44). Moreover, A-tDCS over the DLPFC may also improve working memory in patients with Alzheimer disease (42). Another recent study showed that applying tDCS to the left temporal lobe effectively improved auditory memory of patients with poststroke cognitive impairment (45). To date, no study evaluated the effects of A-tDCS over left temporal lobe on cognitive function in patients with TBI.

Considering the possible cognitive effects of tDCS and the clinical importance of TBI, the purpose of this study is to investigate the early effects of tDCS in patients diagnosed with PPCS exhibiting cognitive deficits in long-term episodic memory, working memory, and executive function. tDCS was chosen instead of rTMS for several reasons, including that tDCS is more suitable for conducting neuropsychological tests (rTMS causes noise and slight discomfort at the stimulation site, which could interfere with patient performance on the tests), that blinding to tDCS is more reliable considering the study design, and that rTMS is expensive. Furthermore, there is a lower risk of seizures related to tDCS than to rTMS, which is contraindicated for patients with an elevated risk of seizures (46).

### Study Purpose and Objectives

The purpose of this study is to determine the early effects of a single session tDCS in patients with MTBI and PPCS with cognitive deficits in long-term episodic memory and executive function (inhibitory control).

### Primary Outcome Measures

The primary hypothesis is that there will be evidence of improvement of patient’s episodic memory and executive function measured by neuropsychological test after the stimulation over the left DLPFC (L-DLPFC) in comparison to the other two types of stimulation [sham stimulation and stimulation over the left temporal cortex (L-TC)].

## Methods and Analysis

### Trial Design

This is a randomized, sham-controlled, crossover trial. All patients will be selected from the outpatient services at the Neurotrauma Clinic of the Hospital das Clínicas of the University of São Paulo Medical School (HCFMUSP). The recruitment period will be from February 2016 to April 2018.

This trial will follow the main Consolidated Standards of Reporting Trials guidelines.

### Participants

Thirty patients with a history of MTBI who are least 18 years of age will be recruited through our outpatient services at the Neurotrauma Clinic. In our institution, all patients sustaining MTBI at the emergency department are advised to come to our outpatient clinic if they have persistent symptoms. Participants must be diagnosed with clinically defined PPCS based upon established criteria for the presence and frequency of three or more current PCS-like symptoms. Those symptoms will be assessed using the Rivermead Post-Concussion Symptoms Questionnaire (RPQ) (47). The inclusion criteria are as follows: (1) a history of MTBI on hospital admission, (2) age between 18 and 60 years, (3) current subjective complaints related to memory and executive function, (4) able to sign an informed consent form, and (5) consent to participate in the study. The exclusion criteria are as follows: (1) outside the age limits, (2) no specific complaints related to memory or executive function, (3) severe symptoms of major depression (Beck Inventory >35), (4) drug addiction, (5) uncontrolled epilepsy, (6) presence of a metallic prosthesis implant, (7) presence of a cochlear implant, (8) intracranial hemorrhage other than subarachnoid hemorrhage on admission CT scan, or (9) unable to sign an informed consent form.

All patients will be informed about tDCS and the experimental protocol, which has been approved by the Ethics Committee of our hospital.

### Recruitment

First the patients will be evaluated by a senior licensed neurosurgeon in the outpatient clinic. Those who present with PCS for a minimum of 3 months will be diagnosed with PPCS. The diagnosis of PPCS will be established using the RPQ (42). Patients will then be referred for a neuropsychological evaluation. Individuals with cognitive impairment related to episodic memory, working memory, and/or executive function will be eligible for the study.

### Procedure

**Figure 1:**
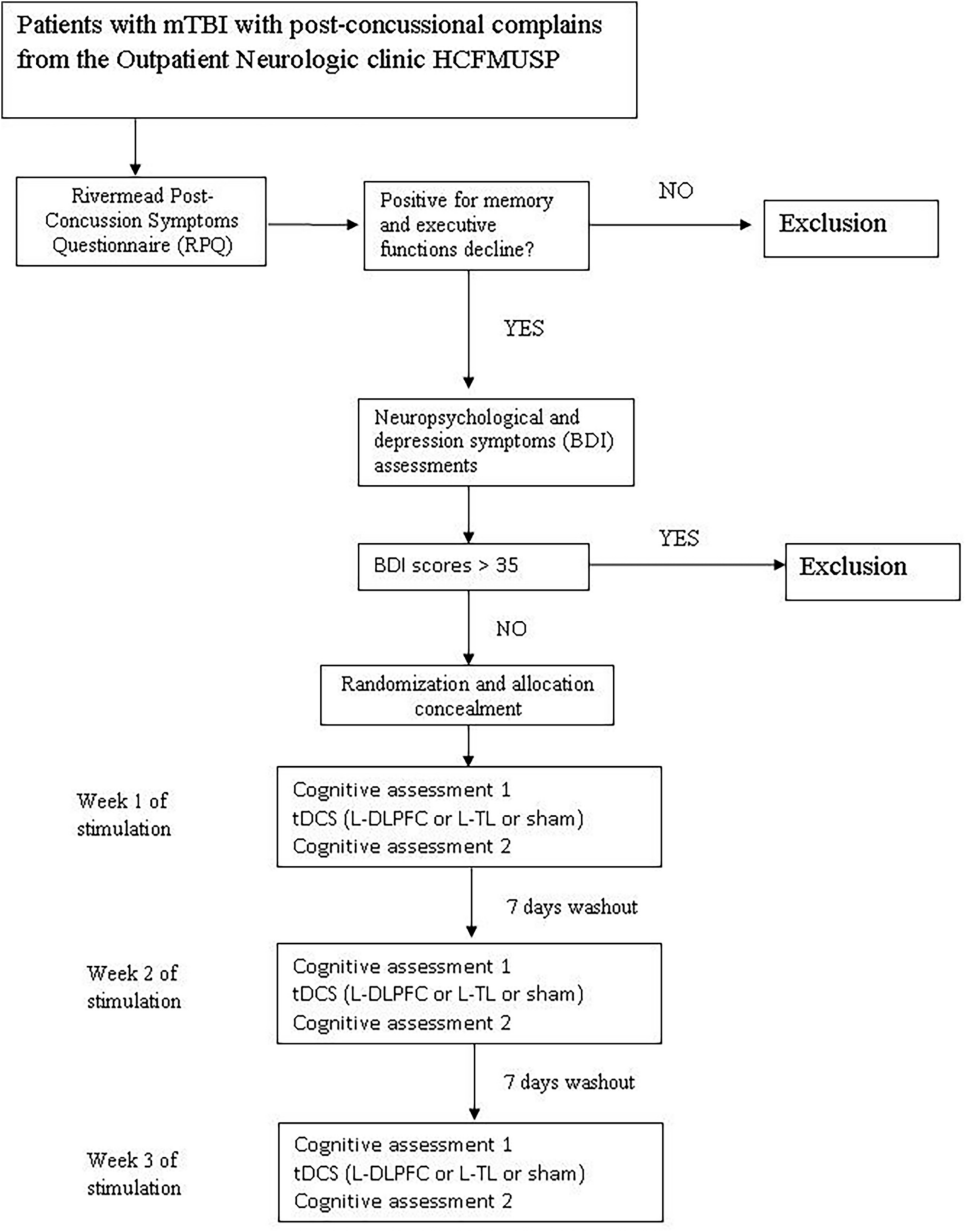
**Fluxogram of the study**.

The study will consist of three phases: (1) a baseline neuropsychological assessment before starting the stimulation sessions; (2) a single tDCS session (L-DLPFC, L-TC, or Sham stimulation) once a week for three consecutive weeks. Figure 1 show the fluxogram of the study.

All the subjects will receive all types of stimulation (L-DLPFC, L-TC, or Sham). The interval between each session will be 7 days to avoid carry-over effects. Because this is a crossover study, all participants will receive all proposed stimulation sessions. A computerized neuropsychological test will be performed before and immediately after each stimulation session.

(A) Frontal stimulation: patients will receive A-tDCS over the L-DLPFC at an intensity of 1.5 mA for 20 min.
(B) Temporal stimulation: patients will receive A-tDCS over the L-TC at an intensity of 1.5 mA for 20 min.
(C) Sham stimulation: patients will receive sham stimulation over the occipital area for only 30 s, after which the current will be turned off automatically without the patient’s knowledge. To guarantee blinding to the stimulation parameters, the A-tDCS electrode will be placed over the occipital region to simulate a study protocol using three different stimulation positions.

### Tolerability and Safety

After each session, patients will be questioned about adverse events. If a major adverse event occurs, the patient will receive medical assistance and further examination and investigation will be provided as needed.

### Instruments

To obtain the necessary data to perform this clinical trial and to analyze the results, the following instruments will be used.

#### Demographic Questionnaire

Information such as age, gender, initial score on the GCS, trauma mechanism, medications in use, and imaging findings will be collected.

#### Rivermead Post-Concussion Symptoms Questionnaire (47)

The RPQ is used to determine the presence and severity of PPCS according to a set of 16 different symptoms commonly found after MTBI. These symptoms are reported by their severity on a scale from 0 (not experienced) to 4 (severe problem). This instrument will be the screening test to identify patients with PPCS.

#### Beck Depression Inventory (BDI) (48)

Brazilian version (49) of the BDI will be used. The BDI is a 21-question multiple-choice self-report inventory designed for individuals aged 13 and over to assess depressive symptoms, such as hopelessness, irritability, guilt, and feeling of being punished, as well as physical symptoms, such as fatigue, weight loss, and lack of interest in sex (31). The BDI ranges from 0 to 63 points. The BDI will be assessed only at baseline.

The above neuropsychological tests will be assessed before and after each tDCS session.

#### Hopkins Verbal Learning Test (50)—Computer Version

This test consists of a list of 12 words. The computer program verbally reproduces the list at a 2-s interstimulus interval. Afterward, the patient is asked to recall as many items as possible in any order. Two additional learning trials are performed, and the delayed recall trial is conducted after a 25-min interval (51).

#### Forward and Backward Digit Span

The computer version of the Wechsler Intelligence Scale for adults (WAIS III) (52) will be used. The digits forward test assesses attention and short-term memory, whereas the digits backward test measures working memory. A random number sequence is presented to the patient at a rate of approximately one number per second. At the end of each sequence, the patient must repeat the digits in the exact sequence (for the forward sequence) in which they were presented or in the opposite order (for the backward sequence). The test is stopped when the patient has consecutive failure on a sequence with the same digit span.

#### Stroop Color–Word Test (53)

The Stroop test measures selective attention, cognitive flexibility, and processing speed. It consists of three cards presented by the examiner. The first card (word card) has 24 rectangles painted in brown, pink, blue, or green; the second card (color card) has 24 words (EACH, NEVER, TODAY, ALL) painted in brown, pink, blue, or green; the third card (color–word card) has 24 words (BROWN, PINK, GREEN, and BLUE) painted with mismatched colors. For each card, the subject is asked to say the name of the color as fast as he/she can. The score is calculated based on the time required to respond for each card.

#### Corsi Block Test (Computerized Version) (53)

This test assesses visual–spatial short-term working memory. This test requires the subject to observe the sequence of blocks “tapped” (illuminated in the computer version) and then repeat the sequence in the same order. The task starts with a short sequence of blocks that gradually increases in number for up to nine blocks. The test measures both the number of correct sequences and the longest sequence remembered.

#### Inhibitory Control Test (ICT)—Computerized Version (54)

This test assesses attention and inhibitory control of action. In this computerized test, the patient is shown a series of letters and is asked to press the backspace of the keyboard when the letter X is followed by the letter Y or if Y is followed by X. X and Y are the target letters; however, during the presentation of the series, other letters are included and serve as distracters. Patients are instructed not to respond to X following X or Y following Y. The ICT is administered as a practice test followed by a series of six similar 2-min trials separated by breaks to allow the subjects to rest. Performance is evaluated as the number of times the patient misses by clicking following an incorrect letter sequence (55).

### Randomization and Blinding

Randomization will be done *via* a computer-produced randomized controlled table. All the 20 patients will be randomized into the three types of stimulation: frontal stimulation, temporal stimulation, and sham stimulation. The neuropsychologists and the patients will be blinded for the type of stimulation performed in each session.

### Electronic Data Collection and Management

Data will be stored in a database developed with the Research Electronic Data Capture system (56), which is hosted on the server of the University of São Paulo. This software developed at Vanderbilt University (TN, USA) is fully web-based and enables electronic data collection, management, and also study process management, while meeting the criteria set by the international policies on data privacy and security in the health sector (57).

### Sample Size Calculation

Most studies which aimed to assess the effects of non-invasive neurostimulation on cognitive function in TBI were case reports or small open labels studies. Moreover, there have been no previous studies comparing the cognitive effects of tDCS stimulation in patients with PPCS. Considering that there were no prior data on the effects of A-tDCS on patients with PPCS using our primary outcome measure, a formal sample size calculation was not possible; thus, we estimated that enrolling 30 patients would be a reasonable approach for an exploratory trial.

### Statistical Analysis

All analysis will be performed using Statistical Package for Social Sciences software version 23.0 for Windows (Prentice Hall, Chicago, IL, USA). A significance level of *p* < 0.05 will be considered for all tests. The quantitative variables will be described using the mean and SDs for normally distributed data or median with inter-quartile range for non-parametric data. The qualitative variables will be presented as absolute and relative frequencies. The five neuropsychological assessments will be summed and averaged to create a composite score. Cohen’s *d* will be calculated to compare the changes in the neuropsychological scores between the groups. An analysis of variance will be used to test whether there is an overall effect of any type of active stimulation on each outcome measure. When appropriate, we will perform *post hoc* paired comparisons using Bonferroni correction for multiple comparisons.

### Ethical Issues

Considering the study’s context and design, there will be minimal risk to patients. Non-invasive neuromodulation techniques follow the ethical criteria for studies involving human participants by respecting the principles of autonomy, beneficence, non-maleficence, and proportionality to ensure that the subject will not be harmed if he/she participates in the study.

Transcranial direct current stimulation can be considered a safe intervention for several reasons: (A) the electric current applied is very low (1–2 mA over an area of 25–35 cm^2^), (B) there is no direct contact between the electrodes and the brain, and (C) the electrodes are embedded in a saline solution, minimizing tissue resistance and avoiding overheating (58–60).

The most common adverse effects observed in safety studies were tingling sensations, itching, mild transient redness of the skin and discomfort on the site of stimulation, moderate fatigue, difficulty concentrating, nausea, and headache. However, these effects were short-lived and were presented at the same frequency between the experimental and placebo groups (60). Patients will be queried after each tDCS session as to whether they experienced adverse effects and how these effects were related to the tDCS treatment. Stimulation sessions have been established by Dr. André Russowski Brunoni, Assistant Professor in the Division of Psychiatry of HC-FMUSP, who will provide any assistance if necessary.

Several advantages of tDCS have been highlighted in clinical practice. These advantages include few side effects that are usually benign, high tolerability, and good potential for efficacy. Notably, it has been emphasized that this technique “has been used in several clinical trials in the last decade and to date, no serious adverse effect has been reported” (58).

## Discussion and Dissemination

This study protocol aims to investigate the neuromodulatory effects of tDCS in patients with a history of MTBI who developed PPCS with a current subjective complaint involving long-term episodic memory, working memory, and executive function. Additionally, the study aims to verify the hypothesis that tDCS exerts pro-cognitive effects in the described population.

Interesting findings have emerged from both clinical trials and neuropsychological studies using tDCS. Knowledge about the cognitive and behavioral functions of brain lesions together with sophisticated neuroimaging techniques have provided major contributions to the fields of neuropsychology and cognitive neuroscience.

The interest in this topic arises from the understanding that neuromodulation techniques can provide causal data that answer questions about the effect of stimulation on cortical structures and specific cognitive functions. The modulatory effect of neuromodulatory stimulation on executive function is of particular interest for understanding the mechanisms underlying the integration of cognition with behavior.

The data that will be obtained in this study may help to provide a step forward for neuropsychology and cognitive neuroscience, as the results will help to reveal brain functioning and the effects derived from interventions. This study also may produce new information regarding the possible pro-cognitive effects of tDCS. Therefore, therapeutic interventions in subsequent studies may be investigated, since the present study will use only a single session of tDCS. We decided to initially study the immediate effects of tDCS in such patients because of the following reasons: (1) we will study a specific population of TBI patients, then, as the first study to evaluate the referred outcomes, we believe that we need to have preliminary data to move forward. (2) We do not know what is the best region to be stimulated, and this trial probably will be able to solve this issue.

This study is expected to initiate a discussion about PPCS thus contributing to the creation of public health policies to treat this underdiagnosed disease. On one hand, PPCS affects a patient’s life in social, work, and cognitive contexts. On the other hand, these patients are poorly supported, and their condition is rarely established based on the findings of imaging exams; this lack of evidence could lead to difficulty in diagnosing and treating PPCS patients.

We believe that tDCS holds great promise. It has been shown in previous studies that tDCS is successful, capable, inexpensive, and safe for use in the treatment of a wide range of neurological conditions. Thus, the application of tDCS might improve the efficiency of different neurorehabilitation techniques and provide further relief to patients suffering from long-term disabilities.

## Ethics Statement

Ethics approval has been obtained from the Ethics Committee for Analysis of Research Projects, Hospital das Clínicas, University of São Paulo (CAPPesq 612.643/14). Written informed consent will be obtained from all participants.

## Author Contributions

RA participated in conception and design of the study, manuscript writing, and its final approval. AB designed the study and helped with data analysis. MO recruited patients, designed the neuropsychological assessment battery, and performed the neuropsychological assessments. AZ recruited patients and designed the neuropsychological assessment battery. MN performed tDCS applications, collected data, reviewed the literature, recruited patients, and contributed to patient follow-up. VG, IN, GB, ML, WP, and AA participated in the conception and design of the study. MT conceived the study and revised the manuscript.

## Conflict of Interest Statement

The authors declare that the research was conducted in the absence of any commercial or financial relationships that could be construed as a potential conflict of interest.
